# Evaluating Short-Term Musculoskeletal Pain Changes in Desk-Based Workers Receiving a Workplace Sitting-Reduction Intervention

**DOI:** 10.3390/ijerph15091975

**Published:** 2018-09-10

**Authors:** Charlotte L. Brakenridge, Yee Ying Chong, Elisabeth A.H. Winkler, Nyssa T. Hadgraft, Brianna S. Fjeldsoe, Venerina Johnston, Leon M. Straker, Genevieve N. Healy, Bronwyn K. Clark

**Affiliations:** 1 School of Public Health, The University of Queensland, Brisbane, QLD 4006, Australia; yeeyingchong@yahoo.com (Y.Y.C.); e.winkler@sph.uq.edu.au (E.A.H.W.); nhadgraft@swin.edu.au (N.T.H.); b.fjeldsoe@sph.uq.edu.au (B.S.F.); g.healy@uq.edu.au (G.N.H.); b.clark3@uq.edu.au (B.K.C.); 2 RECOVER Injury Research Centre, The University of Queensland, Brisbane, QLD 4006, Australia; v.johnston@uq.edu.au; 3 Centre for Urban Transitions, Swinburne University of Technology, Melbourne, VIC 3122, Australia; 4 Baker Heart and Diabetes Institute, Melbourne, VIC 3004, Australia; 5 School of Health and Rehabilitation Sciences, The University of Queensland, Brisbane, QLD 4072, Australia; 6 School of Physiotherapy and Exercise Science, Curtin University, Perth, WA 6102, Australia; l.straker@curtin.edu.au

**Keywords:** office workers, workplace, sitting, sedentary behaviour, musculoskeletal discomfort, musculoskeletal pain, low back pain, trial, activity monitor

## Abstract

This paper explores changes in musculoskeletal pain among desk-based workers over three months of a workplace-delivered, sitting-reduction intervention. Participants (*n* = 153, 46% female; mean ± SD aged 38.9 ± 8.0 years) were cluster-randomized (*n* = 18 work teams) to receive an organizational change intervention, with or without an activity tracker. A modified Nordic Musculoskeletal Questionnaire assessed pain intensity (0–9; none–worst possible) in the neck, upper and lower back, upper and lower extremities, and in total. The activPAL3 (7 days, 24 h/day protocol) measured sitting and prolonged sitting in ≥30 min bouts at work. Mixed models adjusting for cluster and intervention arm examined changes in pain (*n* = 104), and their associations with reductions in sitting and prolonged sitting (h/10 h at work) (*n* = 90). Changes in pain were nonsignificant (*p* ≥ 0.05) and small for total pain (−0.06 [95% CI: −0.27, 0.16]) and for each body area (−0.26 [−0.66, 0.15] for upper back to 0.09 [−0.39, 0.56] for lower back). Sitting reduction was associated with reduced lower back pain (−0.84 [−1.44, −0.25] per hour, *p* = 0.005); other effects were small and non-significant. No substantial average changes in pain were seen; some improvement in lower back pain might be expected with larger sitting reductions. Larger samples and diverse interventions are required for more definitive evidence.

## 1. Introduction

Pain from the musculoskeletal system affects up to 20% of adults worldwide [1], accounting for 21.3% of all years lived with disability globally [2]. The impact of musculoskeletal disorders is particularly highlighted in the workplace setting, where they contribute substantially to annual illness and injury costs and reduced productivity [3]. Interventions that can prevent or reduce the impact of musculoskeletal disorders are therefore of interest to both occupational and public health sectors.

Evidence suggests that neck [4] and lower extremity pain [5] may be associated with sitting for long periods at work, and upper extremity problems [6] may be associated with computer use. Prolonged sitting can also be an aggravating factor for lower back pain when combined with awkward postures (e.g., sitting forward and not upright) or whole-body vibration [7]. Desk-based workers in particular engage in high levels of sitting [8], spending an average of 75% of their work hours sitting [8], and with much of this sitting time accrued in prolonged, unbroken bouts of 30 min or more [8]. The most common musculoskeletal complaints among desk-based workers are neck pain, shoulder pain and lower back pain [9]. The direction of causation (i.e., whether pain affects movement, vice versa, or both) is not well established.

It is possible that reducing sitting may improve musculoskeletal discomfort through the regular interruption of static postures [10] and the reduction of lumbar spine slumped posture that can occur when sitting for prolonged periods [11]. Alternatively, detrimental impacts could occur if sitting is replaced with prolonged periods of static standing, which may harm musculoskeletal health [12]. There is conflicting evidence as to what impact, if any, sitting reduction interventions have on musculoskeletal symptoms [13,14,15]. Reviews of workplace sitting interventions have found improved, worsened or no change in various musculoskeletal outcomes [13,14,15,16]. The reviews have noted the evidence to be particularly inconsistent concerning musculoskeletal pain in different body parts, and the evidence basis involves primarily interventions that use sit–stand workstations. Plausibly, musculoskeletal effects may differ when intervening on sedentary behaviour at work without sit–stand workstations, or outside the workplace, such as in work-disabled populations.

Using data from Stand Up Lendlease—a workplace sitting intervention without modifications to desk configurations, i.e., no sit–stand workstations and no treadmill desks—the present study aimed to add to this underexplored topic. We examined average changes in the intensity of musculoskeletal pain over the intervention overall and in the following body areas (neck, upper extremities, upper back, lower back, and lower extremities). We also assessed whether participants’ changes in pain were associated with their reductions in total and prolonged sitting time at work. Both intervention groups received organizational support and one group also received the LUMOback posture-based activity tracker [17]. The trial’s previous evaluation showed no large or significant changes on average in the workplace sitting outcomes in either group over the short term (three months), with also no large or significant differences between groups [17]. In the groups with and without the tracker, respectively, sitting at work reduced (non-significantly) by 11 min and 4 min (total sitting) and by 6 min and 10 min (prolonged sitting) on average per 10 h workday; however, there was substantial individual variability in change [17].

## 2. Materials and Methods

### 2.1. Study Design and Participants

Stand Up Lendlease was a cluster-randomized trial to reduce desk-based workers’ prolonged sitting time. The trial was conducted in a single property and infrastructure organization from 2014 to 2015, with assessments at baseline, three, and 12 months. To be eligible for the trial, participants had to be ambulatory (i.e., be able to walk at least 10 m), work at least 0.5 full-time equivalent, and at the beginning of the trial: not have a personal activity-permissive workstation at work and not be pregnant. Participants were 153 desk-based office workers from 18 manager-led teams, who were randomized by team to one of two interventions: 1) organizational support only (‘Group ORG’; *n* = 9 teams, 87 participants) or 2) organizational support and a LUMOback activity tracker (‘Group ORG+Tracker’; *n* = 9 teams, 66 participants). The trial was approved by the University of Queensland Behavioural and Social Sciences Ethical Review Committee (approval number 2014000089). The current secondary analyses concerning short-term (three-month) changes in musculoskeletal pain had additional ethical approval from the University of Queensland, School of Public Health Research Ethics Committee (approval number 1719). Both the trial and this study were conducted in accordance with the Declaration of Helsinki. All participants gave written informed consent. Further details on the trial can be found in previous publications [17,18].

### 2.2. Intervention

The organizational support component of the intervention was primarily led by a workplace champion (the Head of Workplace Wellbeing of the organization). The workplace champion delivered an information booklet, welcome email, five fortnightly emails, and workplace health presentations to participants and had ongoing discussions with team managers. The trial had the support of the organization’s senior global executives. The research team also emailed participants’ individual feedback on their sitting, standing, and stepping time as measured via the activPAL monitor (see below) after each assessment, as well as group-level summaries at baseline.

The workplace champion also delivered a LUMOback tracker (LUMO Bodytech, Mountain View, CA, USA) to participants in the ORG+Tracker group. The LUMOback, worn as a belt, measures sitting, standing, sit-to-stand transitions, walking, running, number of steps, posture (distinguished by pelvic tilt angle) and sleep, and provides real-time feedback on these behaviours through a mobile application [19]. The LUMOback also provides real-time prompts when the user is in a poor (slumped) lumbar posture and when the user engages in prolonged periods of sitting time (user-defined, prompts can be set from 15 min to 2 h). Usage of the LUMOback in the Stand Up Lendlease trial was low (median of eight days use in the first three months) and has been reported in more detail in a previous publication [20].

### 2.3. Data Collection

An online questionnaire collected data on participants’ socio-demographics, health, and occupational characteristics at baseline, and musculoskeletal health measures at baseline and three months. At baseline and three months, participants were also asked to wear an activPAL3 activity monitor (PAL Technologies Ltd., Glasgow, Scotland, UK) 24 h per day for 7 days for assessment of sitting and prolonged sitting time as well as physical activity. Over this same timeframe, participants completed electronic diaries that collected times they woke up, went to bed, started and finished work, and removed the monitor.

Due to ethical requirements the data from this study is stored in a folder accessible only to the members of the research team. Participant consent was not given for this data to be shared publicly.

### 2.4. Measures

#### 2.4.1. Participant Characteristics

Participant characteristics measured at baseline via the questionnaire were age, sex, highest level of education completed, current smoking status, knowledge of the health impacts of sitting [21], full-time equivalent work hours, job category (team leader or senior management/other managerial/general staff), job performance [22], work satisfaction [23], job control [23], relationships with supervisor [23], stress [23], and quality of life [24]. Body mass index (BMI, kg/m^2^) was calculated from height and weight. Participants present at measurement sessions had their baseline height measured, without shoes, using a stadiometer (to the nearest 0.1 cm) and weight measured via calibrated electronic scales (to the nearest 0.1 kg). Eleven participants who were absent self-reported their height and weight. Average weekday work hours per work day were calculated from the self-report diaries.

#### 2.4.2. Musculoskeletal Pain

Musculoskeletal pain was evaluated through a modified version of the Nordic Musculoskeletal Questionnaire (NMQ) [25]. The questionnaire collected data regarding musculoskeletal symptoms, and pain intensity in nine body areas: the neck, shoulder, elbow, wrists/hands, upper back, lower back, hips/thighs/buttocks, knees and ankle/feet. The questions were modified to ask about the previous month rather than the previous 12 months as per the original NMQ [26]. Those reporting no symptoms were assigned a pain score of zero, while those who reported having musculoskeletal symptoms were also asked to report how intense pain was on average over the last month, on a scale from zero, which refers to no complaints, to nine, which refers to the pain ‘as bad as it can be’ [27]. The specific questions can be found in the supplementary material of the study protocol [18]. Pain scores were examined as: total pain (i.e., mean pain intensity across all nine areas); neck pain; upper extremity pain (pain in the shoulder, elbow, and wrists/hands); upper back pain; lower back pain; and, lower extremity pain (pain in the knees, ankle/feet, hips/thighs/buttocks). Pain scores in the upper and lower extremities were calculated as the mean of the 0–9 pain scores in the relevant body areas. Alternative measures, based on the maximum 0–9 pain score in the relevant body areas, were highly correlated with the mean-based scores (Pearson’s correlations all >0.9); thus, only the mean-based scores are reported.

#### 2.4.3. Sitting and Prolonged Sitting Time

The activPAL activity monitor was used to collect data on participants’ sitting and activity. This monitor is a reliable and valid tool to measure sitting time and sitting accumulation [28,29]. Monitors were fitted, collected, and downloaded, and activity data were extracted via procedures described elsewhere in detail [17,18]. Briefly, a bespoke SAS program (version ≥9.3) (SAS Institute, Cary, NC, USA) was used to extract the average time per valid day that participants spent sitting at work, engaged in prolonged sitting bouts ≥30 min at work, and engaged in stepping at a moderate–vigorous physical activity (MVPA) level of ≥3 Metabolic Equivalents overall (as per the default device output). Waking hours, wear time, work time, and sleep were identified from diaries, with a lack of movement used to infer sleep and non-wear when diary data were incomplete. For workplace activity data to be considered valid, ≥80% of work time needed to be monitored. For overall activity to be considered valid, the monitor needed to be worn for ≥80% of waking hours, with a further requirement of ≥10 h wear time if waking hours were inferred from movement rather than self-reported.

### 2.5. Sample Size

The sample size for the Stand Up Lendlease trial was selected to provide adequate power for the primary outcomes [18] without reference to secondary outcomes such as musculoskeletal pain. For this study, minimum differences of interest (MDI) were set at ‘moderate’ effect sizes (0.5 SD). Sample size adequacy for the present secondary analysis is evaluated based on whether or not the sample size was adequate to either detect a significant effect, or for non-significant effects, indicate that the true effect in the population is unlikely to be substantial (≥ the MDI), based on the 95% confidence intervals.

### 2.6. Statistical Methods

STATA version 14 (StataCorp LP, College Station, TX, USA) was used for data analysis with statistical significance set at *p* < 0.05 two-tailed. The degree of work-team clustering (intracluster correlations; ICC) was inestimably small (< 0.001) for changes in neck, upper back, lower back and total pain, but was not negligible for upper extremity pain (ICC = 0.040, 95%CI: 0.002, 0.547) and lower extremity pain (ICC = 0.033, 95% CI: < 0.001, 0.802). Therefore, all estimates were adjusted for clustering, either by using mixed models with a random intercept or linearized variance estimation (i.e., ‘survey commands’).

Changes over time in continuous pain scores were tested via mixed models that included terms for time, group (Group ORG/Group ORG + Tracker), a random intercept for cluster (work team), and a residual error term for the repeated measures. Further models that also included a group by time interaction were also examined to test the assumption that the changes over time could reasonably be pooled across the two intervention arms. Associations of short-term (three-month) reductions in sitting time and prolonged sitting time with short-term changes in pain scores were also examined using mixed models, adjusting for group, and with a random intercept for cluster. Missing data were excluded; analyses of changes were limited to those with baseline and three-month data. The sensitivity of the conclusions to assumptions regarding missing data were tested by performing multiple imputation analyses of all randomized participants. Missing data were imputed by chained equations, using *m* = 80 imputations, which was sufficient relative to the fraction of missing information, and including in the imputation models all variables used in the analytic models as well as predictors of missing data. Predictors of missing data were selected as those variables that showed an association with missing data that was either significant at *p* < 0.2 or substantial (odds ratio ≥ 2 or equivalently ≤ 0.5) in ‘survey’ logistic regression models.

## 3. Results

### 3.1. Participant Characteristics

Baseline characteristics of Stand Up Lendlease participants overall and within each intervention arm are shown in Table 1; a participant flow chart is provided in Supplemental Table S1. The 18 work teams each contained 3–14 participants (mean = 8.5). The average (mean ± SD) age was 38.9 ± 8.0 years and BMI was 24.6 ± 3.4 kg/m^2^. Just under half of participants were female (*n* = 70, 45.8%). Most participants worked in managerial roles with a minority of general staff (48/153, 31.4%). Nearly all participants worked full time (136/145, 93.8%), on average working for 9.8 ± 1.1 h per workday on weekdays. Baseline pain levels ranged from an average of 0.7 ± 1.1 and 0.7 ± 1.0 for pain in the lower and upper extremities, respectively, to 1.4 ± 2.0 in the lower back and 1.5 ± 2.1 in the neck, with total pain averaging 1.1 ± 1.1. Of the 135 participants reporting on their baseline pain, most reported pain (*n* = 107, 79.3%), typically in multiple areas (*n* = 77, 57.0%) with a few reporting pain in all five areas (*n* = 7, 5.2%; Supplemental Table S2). Participants most commonly reported pain in the upper extremities (*n* = 62, 45.9%), lower extremities (*n* = 57, 42.2%), neck (*n* = 56, 41.5%) or lower back (*n* = 54, 40.0%) and some reported upper back pain (*n* = 39, 28.9%).

Participants who were missing data on changes in musculoskeletal pain and workplace activity (due to dropping out or skipping an assessment) differed significantly from their counterparts who provided full data (Supplemental Table S3) in terms of having higher job control (*p* = 0.049) and less awareness of the health benefits of sitting less, as indicated by lower sitting knowledge scores (*p* = 0.037). The odds of missing data also tended to be higher in men (than women), in those with managerial jobs (than general staff), with longer weekday work hours, less sitting at work, and with lower initial pain scores.

### 3.2. Short-Term Changes in Musculoskeletal Pain Intensity

Changes over time in musculoskeletal pain intensity did not differ significantly between the intervention arms (*p* = 0.500–0.740); thus, results are reported pooled (Table 2). In both the completer analyses and after accounting for missing data, changes in pain were all small and not statistically significant. Confidence intervals contained only small changes in either direction, except in the case of upper back pain, where the confidence interval contained moderate effects and thus a moderate reduction could not be ruled out as unlikely. In study completers, changes ranged from a small nonsignificant decrease in upper back pain (−0.26, 95% CI: −0.66, 0.15, *p* = 0.209) to a small nonsignificant increase in lower back pain (0.09, 95%: CI −0.39, 0.56, *p* = 0.719) with total pain tending to decrease to a very small degree (−0.06, 95% CI: −0.27, 0.16, *p* = 0.597). Changes were sometimes attenuated after accounting for missing data. In the multiple imputation analyses, changes ranged from a small, nonsignificant reduction in upper back pain (−0.14, 95% CI: −0.58, 0.30, *p* = 0.528) to a small nonsignificant increase in lower back pain (0.06, 95% CI: −0.45, 0.57, *p* = 0.816), with no change observed in total pain (−0.00, 95% CI: −0.25, 0.25, *p* = 0.999).

### 3.3. Associations of Workplace Sitting Reduction with Changes in Musculoskeletal Pain

Table 3 shows the associations of reductions in sitting and prolonged sitting time at work with changes in musculoskeletal pain. There were significant small-to-moderate reductions in lower back pain with each one-hour per day reduction in sitting time at work (*b* = −0.84, 95% CI: −1.44, −0.25, *p* = 0.005 in study completers and *b* = −0.61, 95% CI: −1.22, −0.01, *p* = 0.047 in multiple imputation analyses). Other pain changes were not significantly associated with reductions in workplace sitting. Further, confidence intervals indicated that only small effect sizes were likely, with the sole exception of upper back pain, where a moderate increase may have been missed in the completer analysis (*b* = 0.33, 95% CI: −0.20, 0.86, *p* = 0.227). The significant association of sitting reduction with lower back pain changes did not appear to be explained by age, sex, BMI, and other potential confounders (*b* = −0.81, 95% CI: −1.44, −0.18, *p* = 0.012 after adjustment; Supplemental Table S4). Further correcting for factors likely to impact sitting reductions attenuated the estimate only slightly (*b* = −0.75, 95% CI: −1.37, −0.13, *p* = 0.017; Supplemental Table 4). In both the completer and multiple imputation analyses, each one-hour reduction in daily prolonged sitting time at work showed only small and non-significant associations with changes in musculoskeletal pain. The largest association was a borderline significant reduction in lower back pain with each hour reduction in prolonged sitting (*b* = −0.39, 95% CI: −0.79, 0.00, *p* = 0.050). This association was attenuated somewhat after accounting for missing data (*b* = −0.30, 95% CI: −0.70, 0.09, *p* = 0.134).

## 4. Discussion

This study evaluated the short-term (three-month) effect of an intervention to reduce sitting time on the intensity of musculoskeletal pain among desk-based workers. Only small, nonsignificant changes in musculoskeletal pain were observed on average, with the overall tendency being more towards reductions rather than increases. None of the pain outcomes were increased significantly or substantially in response to sitting or prolonged sitting reduction at work. Rather, lower back pain was significantly reduced by a small amount with reductions in workplace sitting time. While the sample size was only sufficient to make conclusive statements regarding moderate effects (0.5 SD) and not small effects, none of the results supported that pain increased in response to the intervention, or to reductions in workplace sitting or prolonged sitting time over the short term.

Plausible mechanisms suggest that improving postural variability, by breaking up long periods of prolonged sitting with ergonomically sound alternatives, should aid desk-workers’ musculoskeletal health [10,11]. However, reviews of the evidence regarding the impact of sitting-reduction interventions on musculoskeletal health [14,15] have indicated that the evidence is insufficient to draw conclusions, with mostly low-quality evidence [14,15] and with inconsistencies between interventions and areas of the body [16]. While concerns have been raised over potential harms of reducing sitting by replacing it with prolonged standing [30], importantly, the evidence outlined in the reviews indicates the types of interventions that have been conducted (typically involving the use of sit–stand workstations and encouraging regular posture changes) show more evidence of improving than worsening musculoskeletal health [13,15,16]. While somewhat inconsistent with the review findings, the lack of any sizeable average improvements over three months in musculoskeletal pain in Stand Up Lendlease was not unexpected given the lack of any large or significant average improvements in workplace sitting or prolonged sitting. Furthermore, depending on the pain outcome, anywhere between approximately 20% and 70% of participants began pain-free with no opportunity for measurable improvement. While it is possible that three months of intervention was too short to improve musculoskeletal pain, this seems unlikely given that other interventions, some as short as five days, have observed pain reductions [31].

The size of the behaviour changes achieved forms only a partial explanation for the lack of any substantial improvement in our present study. When exploring dose-response by testing associations between the extent of workplace sitting reduction and musculoskeletal improvement, findings suggested that a greater degree of workplace sitting reduction could lead to greater reductions to lower back pain. The same was not the case for pain in the other body areas (i.e., neck, upper and lower extremities and upper back), which showed no sizeable or significant association with extent of change in workplace sitting or prolonged sitting time. It is difficult to ascertain whether or not the specific salience of lower back pain relative to other forms of pain in Stand Up Lendlease is likely to be the case for other similar interventions. A recent review by Agarwal et al [16] noted the inconsistencies across body areas, with some interventions, like ours, suggestive that reducing sitting may improve lower back discomfort [31,32] and others suggesting that discomfort is improved in other areas (specifically, the neck and shoulders) but not in the lower back [33,34].

Varying results across interventions are not entirely unexpected, since sitting-reduction interventions have tended to focus on general worker samples rather than clinically homogeneous subgroups (e.g., those with a specific diagnosis of a musculoskeletal disorder) who may respond more uniformly to intervention. Further, the interventions delivered are also not homogeneous, with workplaces and/or workers generating their own strategies for change [35] and involving variable [32] or unmeasured [33,34] ‘doses’ in terms of sit–stand desk usage. In Stand Up Lendlease, sitting-reduction strategies varied between individuals, with the four strategies with the most increase in usage being standing meetings, walking meetings, standing on the phone, and walking to a printer further away [36]. Different strategies could have plausibly disparate musculoskeletal sequelae, from simple considerations, such as whether sitting is replaced with standing or stepping, to an array of unreported factors concerning how ergonomically sound workers’ previous behaviours were, and their new behaviours become.

### Strengths and Limitations

A strength of this study was that in addition to reporting on average changes in pain during the intervention, dose-response was also explored by examining how participants’ changes in high-quality, objective measures of sitting time related to their changes in musculoskeletal pain over the course of the intervention. Previous trials have tended to omit the dose-response aspect [33] or test dose-response only in relation to pain presence/absence [37], which requires much larger samples for definitive results relative to continuous measures. While biases generated by a relationship between participant pain and the propensity to stay in the study and undergo assessments can be an issue in general, we verified our general conclusions were consistent across completers and multiple imputation analyses regarding whether or not effects were statistically significant and of a magnitude consistent with our MDI. In our study, the size of the associations between sitting reduction and lower back pain changes are potentially overstated in completer analyses by ~40%; it would be good to see more of such sensitivity analyses in the extant literature. The lack of a control group is a limitation, particularly in ascertaining whether any changes, if observed, were produced by the intervention rather than other factors (such as seasonality). Further, in linking activity changes with pain changes, we primarily focused on dose-response interpretations. However, reverse causation is still plausible, even within an intervention, as increases in pain (arising from any source) could limit the extent of activity change [38], or the causation could be bidirectional [38]. While continuous scores of pain intensity are commonly used to measure pain, the validity and reliability of our measures have not been tested (to our knowledge), and a lack of validity could result in a failure to detect changes. Also, the models displayed some non-normality, which could not be rectified via transformations. Being a secondary analysis, this study was not powered a priori concerning the hypotheses tested. Most confidence intervals around nonsignificant effects indicated that the true effect size was unlikely to be substantial (≥0.5 SD). However, the study could not provide definitive results regarding the potential association between lower back pain and reductions in prolonged sitting time, nor between upper back pain and reductions in total sitting time. Also, too few participants re-consented into the study to evaluate long-term changes over 12 months. Results obtained should be interpreted with caution due to the exploratory nature of this study and also should not be generalized to interventions that promote substantially different sitting-reduction strategies (e.g., by providing workspace modification).

## 5. Conclusions

In the Stand Up Lendlease trial, no large or significant increases or decreases in average musculoskeletal pain intensity were observed over three months in total pain, and pain across five body areas: the neck, upper extremities, upper back, lower back, and lower extremities. A reduction in sitting time at work was significantly associated with small reductions in lower back pain only. While not definitive, this suggests that similar sitting-reduction interventions that are more effective at achieving behaviour change might see an improvement in this pain area that we failed to observe, while the same is less likely to be the case for the other body areas. Alternatively, it could be the case that pain increases (experienced for any reason) limited the degree of sitting-reduction achieved, and better intervention messaging that is sensitive to participants’ pain fluctuations may be important to help pain-prone individuals achieve change. Future studies using larger sample sizes, over a longer duration, with more regular data collections, are needed to determine the long-term relationships between sitting-time reduction and musculoskeletal pain. Ideally, greater detail regarding participant movements (e.g., via video recording) should also be collected to help better understand why changes occur in specific areas of the body, and to refine intervention design to maximize benefits and prevent harms.

## Figures and Tables

**Table 1:** Baseline characteristics of Stand Up Lendlease participants.

Characteristics	ORG		ORG + Tracker		Overall	
	*n*	*n*(%) or mean ± SD ^1^	*n*	*n*(%) or mean ± SD ^1^	*n*	*n*(%) or mean ± SD ^1^
Age, years	80	40.0 ± 8.0	63	37.6 ± 7.7	143	38.9 ± 8.0
Sex, % female	87	35 (40.2%)	66	35 (53.0%)	153	70 (45.8%)
Body mass index, kg/m^2^	68	25.0 ± 3.4	50	24.1 ± 3.4	118	24.6 ± 3.4
Job category	87		66		153	
Senior leader/team leader		17 (19.5%)		8 (12.1%)		25 (16.3%)
Other managerial		42 (48.3%)		38 (57.6%)		80 (52.3%)
General staff		28 (32.2%)		20 (30.3%)		48 (31.4%)
Full-time equivalent, %Full-time	81	77 (95.1%)	64	59 (92.2%)	145	136 (93.8%)
Weekday work hours, h/workday	86	9.9 ± 1.2	63	9.6 ± 1.0	149	9.8 ± 1.1
Education, %≥university	81	67 (82.7%)	63	54 (85.7%)	144	121 (84.0%)
Smoking, %currently smoke	77	8 (10.4%)	58	5 (8.6%)	135	13 (9.6%)
Sitting knowledge (1–5) ^2^	76	3.9 ± 0.6	58	3.9 ± 0.5	134	3.9 ± 0.5
Physical quality of life (0–100) ^2^	72	50.9 ± 8.0	56	51.8 ± 6.8	128	51.3 ± 7.5
Mental quality of life (0–100) ^2^	72	44.0 ± 11.9	56	46.1 ± 10.3	128	44.9 ± 11.2
Stress (1–10) ^3^	75	6.6 ± 2.1	56	6.2 ± 2.5	131	6.5 ± 2.3
Job performance (1–10) ^2^	75	7.5 ± 0.9	56	7.7 ± 0.9	131	7.6 ± 0.9
Job control (1–10) ^2^	74	6.8 ± 1.8	55	6.9 ± 1.9	129	6.8 ± 1.8
Supervisor relations (1–10) ^2^	75	7.3 ± 1.7	56	6.8 ± 1.8	131	7.1 ± 1.8
Work satisfaction (1–10) ^2^	75	6.3 ± 1.4	56	6.6 ± 1.6	131	6.4 ± 1.5
Total pain (0–9) ^3^	77	1.1 ± 1.1	58	1.0 ± 1.1	135	1.1 ± 1.1
Neck pain (0–9) ^3^	77	1.4 ± 2.0	58	1.7 ± 2.2	135	1.5 ± 2.1
Upper extremity pain (0–9) ^3^	77	0.7 ± 1.1	58	0.6 ± 0.9	135	0.7 ± 1.0
Upper back pain (0–9) ^3^	77	1.0 ± 1.8	58	1.0 ± 1.7	135	1.0 ± 1.7
Lower back pain (0–9) ^3^	77	1.5 ± 2.1	58	1.3 ± 2.0	135	1.4 ± 2.0
Lower extremity pain (0–9) ^3^	77	0.8 ± 1.1	58	0.6 ± 1.0	135	0.7 ± 1.1
Work sitting, h/10 h workday	84	7.3 ± 1.0	62	7.6 ± 0.9	146	7.4 ± 1.0
Work prolonged sitting, h/10 h workday	84	4.1 ± 1.4	62	4.2 ± 1.5	146	4.2 ± 1.4
MVPA ^4^, min/16 h day	85	77.5 ± 22.7	64	81.3 ± 22.9	149	79.2 ± 22.9

^1^ SD adjusted for clustering by linearized variance estimation. ^2^ Higher scores are favourable. ^3^ Lower scores are favourable. ^4^ MVPA = moderate–vigorous physical activity.

**Table 2:** Mean changes in musculoskeletal pain intensity over three months of the Stand Up Lendlease sitting-reduction intervention.

Pain Intensity (0–9, None–Worst)	Completer Analyses (*n* = 104)				Multiple Imputation (*n* = 153)	
	ORG + Tracker versus ORG		Pooled Estimate		Pooled Estimate	
	Difference (95% CI)	*p*	Mean Change (95% CI)	*p*	Mean Change (95% CI)	*p*
Total pain	−0.08 (−0.52, 0.36)	0.721	−0.06 (−0.27, 0.16)	0.597	−0.00 (−0.25, 0.25)	0.999
Neck pain	−0.30 (−1.21, 0.60)	0.511	−0.08 (−0.52, 0.37)	0.735	0.02 (−0.47 0.52)	0.928
Upper extremity pain	0.08 (−0.37, 0.52)	0.740	−0.03 (−0.25, 0.19)	0.796	0.04 (−0.21, 0.28)	0.752
Upper back pain	−0.19 (−1.02, 0.63)	0.649	−0.26 (−0.66, 0.15)	0.209	−0.14 (−0.58, 0.30)	0.528
Lower back pain	0.17 (−0.79, 1.13)	0.730	0.09 (−0.39, 0.56)	0.719	0.06 (−0.45, 0.57)	0.816
Lower extremity pain	−0.15 (−0.59, 0.29)	0.500	−0.01 (−0.23, 0.20)	0.907	0.01 (−0.21, 0.23)	0.904

Table presents difference between groups in mean changes, and pooled estimates of mean change (adjusted for group), from linear mixed models that adjust for cluster (random intercept).

**Table 3:** Associations of reductions in workplace sitting and prolonged sitting with changes in musculoskeletal pain intensity over three months of intervention.

Pain Intensity Outcome (0–9, None–Worst)	Model	Sitting Reduction (h/10 h at Work)		Prolonged Sitting Reduction (h/10 h at Work)	
		*b* (95% CI)	*p*	*b* (95% CI)	*p*
Total pain	Completer	−0.04 (−0.32, 0.25)	0.805	−0.04 (−0.22, 0.15)	0.697
	MI	−0.02 (−0.29, 0.25)	0.870	−0.02 (−0.22, 0.17)	0.803
Neck pain	Completer	0.14 (−0.43, 0.72)	0.626	0.07 (−0.31, 0.45)	0.715
	MI	0.10 (−0.49, 0.69)	0.742	0.02 (−0.38, 0.42)	0.923
Upper extremity pain	Completer	0.11 (−0.16, 0.39)	0.421	0.00 (−0.19, 0.18)	0.971
	MI	0.13 (−0.14, 0.41)	0.340	0.01 (−0.17, 0.19)	0.911
Upper back pain	Completer	0.33 (−0.20, 0.86)	0.227	0.11 (−0.24, 0.46)	0.525
	MI	0.21 (−0.33, 0.75)	0.440	0.10 (−0.24, 0.44)	0.568
Lower back pain	Completer	−0.84 (−1.44, −0.25)	0.005	−0.39 (−0.79, 0.00)	0.050
	MI	−0.61 (−1.22, −0.01)	0.047	−0.30 (−0.70, 0.09)	0.134
Lower extremity pain	Completer	0.07 (−0.21, 0.35)	0.611	0.01 (−0.17, 0.20)	0.873
	MI	0.10 (−0.17, 0.36)	0.471	0.03 (−0.14, 0.19)	0.749

Table presents unstandardized regression coefficient (b) and 95% confidence intervals for a reduction of 1 h per 10 h workday in sitting or prolonged sitting time at work. Estimates are obtained from linear mixed models, adjusting for intervention arm (fixed effect) and clustering (random intercept) in either completer analyses (*n* = 90) or multiple imputation (MI) analyses (*n* = 153).

## Supplementary Materials

The following are available online at http://www.mdpi.com/1660-4601/15/9/1975/s1, Table S1: Participant flow chart., Table S2: Prevalence of pain at baseline and three months in all Stand Up Lendlease participants., Table S3: The odds of missing data on three-month pain and activity changes by baseline characteristics in all Stand Up Lendlease participants at baseline., Table S4: Associations of sitting time reductions (h/10 h at work) with changes in lower back pain scores (completer analyses).
