# Supplementary material for: Evaluating Short-Term Musculoskeletal Pain Changes in Desk-Based Workers Receiving a Workplace Sitting-Reduction Intervention
Source: Int J Environ Res Public Health. 2018 Sep 10;15(9):1975. doi: 10.3390/ijerph15091975 (PMC6164528; doi:10.3390/ijerph15091975)
Supplement: Supplementary file 1 [file ijerph-15-01975-s001.pdf]

International Journal of
Environmental Research and Public Health

MDPI

Supplementary Materials

Evaluating Short-Term Musculoskeletal Pain Changes in Desk-Based Workers Receiving a Workplace Sitting-Reduction Intervention

Charlotte L. Brakenridge, Yee Ying Chong, Elisabeth A.H. Winkler, Nyssa T. Hadgraft, Brianna S. Fjeldsoe, Venerina Johnston, Leon M. Straker, Genevieve N. Healy and Bronwyn K. Clark

Supplemental Table S1. Participant flow chart.

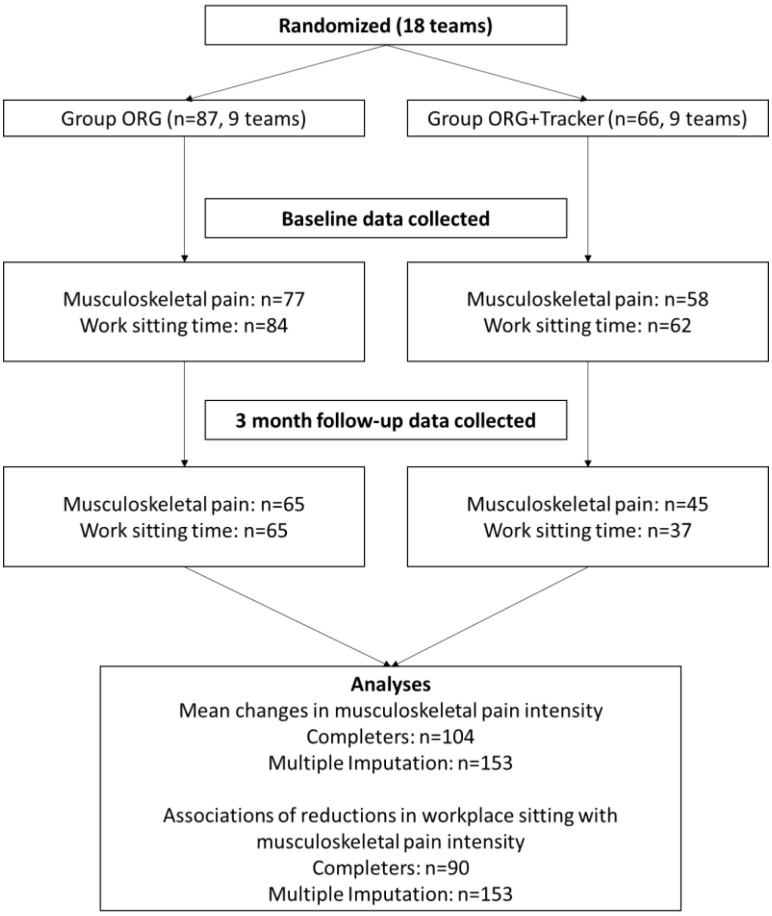

Supplemental Table S2. Prevalence of pain at baseline and three months in all Stand Up Lendlease participants.

	Baseline (*n* = 135)	3 months (*n* = 110)
Neck pain	56 (41.5%)	45 (40.9%)
Upper extremity pain	62 (45.9%)	45 (40.9%)
Upper back pain	39 (28.9%)	24 (21.8%)
Lower back pain	54 (40.0%)	41 (37.3%)
Lower extremity pain	57 (42.2%)	49 (44.5%)
Number of areas with pain (/5), n(%)		
0	28 (20.7%)	26 (23.6%)
1	30 (22.2%)	27 (24.5%)
2	28 (20.7%)	18 (16.4%)
3	21 (15.6%)	24 (21.8%)
4	21 (15.6%)	6 (5.5%)
5	7 (5.2%)	9 (8.2%)

Supplemental Table S3. The odds of missing data on three-month pain and activity changes by baseline characteristics in all Stand Up Lendlease participants at baseline.

Characteristic	*n*	Odds Ratio (95% CI)	*p*
Intervention arm (ORG + Tracker vs ORG)	153	1.52 (0.66, 3.53)	0.305
Age, per 10 years	143	1.08 (0.65, 1.78)	0.754
Sex (female vs male)	153	0.53 (0.26, 1.05)	0.065
Body mass index, kg/m^2	118	1.02 (0.87, 1.19)	
Job category	153		0.061
Senior leader/team leader	25	1 (ref)	
Other managerial	80	1.34 (0.36, 4.94)	0.643
General staff	48	0.38 (0.08, 1.87)	0.216
Weekday work hours, h/workday	149	1.45 (0.92, 2.27)	0.101
Education (university vs <university)	144	1.15 (0.44, 2.99)	0.760
Currently smoke (yes vs no)	135	1.82 (0.55, 6.03)	0.302
Sitting knowledge (1-5), per unit	134	0.50 (0.26, 0.95)	0.037
Physical quality of life (0-100), per 10 units	128	0.87 (0.57, 1.32)	0.495
Mental quality of life (0-100), per 10 units	128	1.14 (0.88, 1.47)	0.311
Stress (1-10), per unit	131	1.03 (0.91, 1.17)	0.590
Job performance (1-10), per unit	131	1.05 (0.66, 1.64)	0.839
Job control (1-10), per unit	129	1.21 (1.00, 1.47)	0.049
Supervisor relations (1-10), per unit	131	1.02 (0.79, 1.30)	0.898
Work satisfaction (1-10), per unit	131	1.12 (0.73, 1.72)	0.570
Total pain (0-9), per unit	135	0.71 (0.49, 1.03)	0.069
Work sitting, per h/10h workday	146	0.78 (0.55, 1.12)	0.162
Work prolonged sitting, per h/10h workday	146	0.85 (0.62, 1.16)	0.284
MVPA, per 10 min/16h day	149	1.03 (0.90, 1.18)	0.627

Table presents odds ratio (OR) and 95% confidence interval (CI) from logistic regression models, using linearized variance estimation ('survey commands') to correct for clustering. MVPA = moderate-vigorous physical activity.

Supplemental Table S4. Associations of sitting time reductions (h/10 h at work) with changes in lower back pain scores (completer analyses).

Model	Adjusts for	*b* (95% CI)	*p*
A. 'Unadjusted' [a]	-	-0.84 (-1.43, -0.25)	0.005
B. Minimally adjusted [a]	age, sex (male/female), BMI category (normal/underweight, overweight/obese, missing)	-0.84 (-1.44, -0.23)	0.007
C. Confounder adjusted [a]	age, sex, BMI category (normal/underweight, overweight/obese, missing), weekday work hours, full time equivalency (1.0 / <1.0), physical quality of life, mental quality of life	-0.81 (-1.44, -0.18)	0.012
D. Further adjusted [a,b]	age, sex, BMI category (normal/underweight, overweight/obese, missing), MVPA, mental quality of life, physical quality of life, job control score, work satisfaction score, desired sitting (over half/under half), current smoker (yes/no)	-0.75 (-1.37, -0.13)	0.017

[a] All estimates are from mixed models that adjust for clustering (random intercept), and intervention arm (fixed effect).

[b] Adjusted for age, sex, BMI category and variables significant at $p<0.2$ out of potential confounders (MVPA, mental quality of life, physical quality of life, weekday work hours, full time equivalency [1.0 / <1.0]) and potential influencers of uptake of the behavioural messages (education, current smoker [yes/no], job control score, work satisfaction score, preferred time spent sitting at work [over/under 50%], current smoker [yes/no], job performance score, supervisor relations score, work satisfaction score, sitting knowledge score, and stress.

© 2018 by the authors. Submitted for possible open access publication under the terms and conditions of the Creative Commons Attribution (CC BY) license (http://creativecommons.org/licenses/by/4.0/).
